# Similarities in Hypertension Status but Differences in Mortality Risk: A Comparison of 2017 ACC/AHA and 2018 Chinese Hypertension Guidelines

**DOI:** 10.3389/fcvm.2022.784433

**Published:** 2022-02-21

**Authors:** Kangyu Chen, Hao Su, Qi Wang, Zhenqiang Wu, Rui Shi, Fei Yu, Ji Yan, Xiaodan Yuan, Rui Qin, Ziai Zhou, Zeyi Hou, Chao Li, Tao Chen

**Affiliations:** ^1^Department of Cardiology, The First Affiliated Hospital of USTC, Division of Life Sciences and Medicine, University of Science and Technology of China, Hefei, China; ^2^Department of Geriatric Medicine, The University of Auckland, Auckland, New Zealand; ^3^Heart Rhythm Centre, The Royal Brompton and Harefield National Health Service Foundation Trust, National Heart and Lung Institute, Imperial College London, London, United Kingdom; ^4^Department of Health Education, Affiliated Hospital of Integrated Traditional Chinese and Western Medicine, Jiangsu Province Academy of Traditional Chinese Medicine, Nanjing University of Chinese Medicine, Nanjing, China; ^5^Department of Epidemiology and Health Statistics, School of Public Health, Xi'an Jiaotong University Health Science Centre, Xi'an, China; ^6^Department of Public Health, Policy and Systems, Institute of Population Health, The University of Liverpool, Liverpool, United Kingdom

**Keywords:** blood pressure, mortality, hypertension, premature mortality, cohort study

## Abstract

**Background:**

Few studies investigated the concordance in hypertension status and antihypertensive treatment recommendations between the 2018 Chinese Hypertension League (CHL) guidelines and the 2017 American College of Cardiology (ACC)/American Heart Association (AHA) guidelines and assessed the change of premature mortality risk with hypertension defined by the ACC/AHA guidelines.

**Methods:**

We used the baseline data of the China Health and Retirement Longitudinal Study (CHARLS) to estimate the population impact on hypertension management between CHL and ACC/AHA guidelines. Mortality risk from hypertension was estimated using the data from China Health and Nutrition Survey (CHNS). Cox proportional hazards model was used to estimate the hazard ratios (HRs) and their 95% confidence intervals(CIs).

**Results:**

Among 13,704 participants analyzed from the nationally representative data of CHARLS, 42.64% (95% CI: 40.35, 44.96) of Chinese adults were diagnosed by both CHL and ACC/AHA guidelines. 41.25% (39.17, 43.36) did not have hypertension according to either guideline. Overall, the concordance in hypertension status was 83.89% (81.69, 85.57). A high percentage of agreement was also found for recommendation to initiate treatment among untreated subjects (87.62% [86.67, 88.51]) and blood pressure (BP) above the goal among treated subjects (71.68% [68.16, 74.95]). Among 23,063 adults from CHNS, subjects with hypertension by CHL had a higher risk of premature mortality (1.75 [1.50, 2.04]) compared with those without hypertension. The association diminished for hypertension by ACC/AHA (1.46 [1.07, 1.30]). Moreover, the excess risk was not significant for the newly defined Grade 1 hypertension by ACC/AHA (1.15 [0.95, 1.38]) when compared with BP <120/80 mmHg. This contrasted with the estimate from CHL (1.54 [1.25, 1.89]). The same pattern was observed for total mortality.

**Conclusions:**

If ACC/AHA guidelines were adopted, a high degree of concordance in hypertension status and initiation of antihypertensive treatment was found with CHL guidelines. However, the mortality risk with hypertension was reduced with a non-significant risk for Grade 1 hypertension defined by the ACC/AHA.

## Introduction

In 2017, the American College of Cardiology/American Heart Association (ACC/AHA) released an updated blood pressure (BP) guidelines and reduced the diagnostic threshold for hypertension to systolic blood pressure (SBP)/diastolic blood pressure (DBP) ≥130/80 mmHg (1). This contrasts with the 2018 Chinese Hypertension League (CHL) BP guidelines and the 2018 European Society of Cardiology (ESC)/European Society of Hypertension (ESH) BP guidelines, where hypertension is diagnosed based on a threshold of ≥140/90 mmHg (2, 3). This new change substantially increases the prevalence and number of subjects with hypertension and extensively revised the treatment recommendations and goal of antihypertensive treatment (4–7). However, few of them investigated the concordances in hypertension management between 2018 CHL and 2017 ACC/AHA. This is of particular interest for the treatment recommendations as the ACC/AHA employs a different strategy to guide the antihypertensive medication treatment through predicted cardiovascular disease (CVD) risk in conjunction with BP (1).

Several studies have assessed the impact of a lower BP threshold on the risk of a cardiovascular event and/or all-cause mortality (8–12). However, a few of them compared the effect of different BP thresholds (e.g., from ACC/AHA and Chinese guidelines) simultaneously. Also, there are uncertainties as to whether either definition of hypertension is associated with premature mortality, which is a very important index for public health surveillance (13). Although some studies demonstrated the positive associations between hypertension (i.e., ≥140/90 mmHg) and premature mortality (14–16), few of them detailed the relationship of premature mortality with different hypertension categories (e.g., stage 1 hypertension) and did not include SBP or DBP to evaluate their specific associations with premature mortality.

In our analysis, we aim to (1) compare the agreement on the definition of hypertension, treatment initiation recommendation, and control status of hypertension between 2017 ACC/AHA and 2018 CHL guideline; and (2) determine whether hypertension or different hypertension categories, as defined by either 2017 ACC/AHA or 2018 CHL guideline, is associated with premature mortality.

## Methods

### Study Populations

We used the baseline data of China Health and Retirement Longitudinal Study (CHARLS) (2011–2012) to estimate the population-level impact of utilizing 2017 ACC/AHA on the prevalence or number of subjects with hypertension, recommendation for antihypertensive treatment, and intensification of therapy, in comparison with the 2018 CHL guidelines. The profile and data quality of this study has been described elsewhere (17). Briefly, CHARLS is a nationally representative survey for 17,708 subjects aged 45 years and older in China and was conducted from 2011 to 2012. The participants were selected through a multistage probability sampling method and can be weighted to obtain national estimates.

To further explore the mortality risk of different subgroups of hypertension defined by ACC/AHA and CHL guidelines, we analyzed the data from China Health and Nutrition Survey (CHNS). CHNS started in 1989 from 9 China provinces that varied substantially in geography, economic development, and followed up every 2–4 years (i.e., in 1989, 1991, 1993, 1997, 2000, 2004, 2006, 2009, and 2011) (18). Since the conflicting results have been repeatedly reported between BP and all-cause mortality among the elderly population (19, 20) and disease burden from early death is relevant to young and middle-aged adults, we only included participants aged 18–75 years with the key information available (e.g., measurements of BP).

The CHARLS and CHNS obtained each participant's written informed consent and were approved by institutional review board of Peking university, and the University of North Carolina at Chapel Hill and the National Institute for Nutrition and Health, Chinese Center for Disease Control and Prevention, respectively. This analysis has been approved by the Human Research Ethics Committee of the Xi'an Jiaotong University Health Science Center (No: 2021-6).

### BP Measurement and Definition

Seated SBP and DBP for each participant were taken using calibrated mercury sphygmomanometers in CHNS or the attended automated BP device (Omron model HEM-7200) in CHARLS following the standardized procedure of each study. The mean value of 3 available BP measurements was used in the current analysis.

The definitions of hypertension, initiation of antihypertensive medication, and BP goals referred to the 2017 ACC/AHA and the 2018 CHL guidelines are displayed in Appendix Table 1 (Supplementary Material). According to the 2018 CHL guidelines, participants were classified into five categories: Normal (untreated SBP <120 mmHg and DBP <80 mmHg); High normal (untreated SBP 120–139 mmHg and/or DBP 80–89 mmHg); Grade 1 hypertension (SBP 140–159 mmHg and/or DBP 90–99 mmHg); Grade 2 hypertension (SBP 160–179 mmHg and/or DBP 100–109 mmHg); Grade 3 hypertension (SBP ≥180 mmHg and/or DBP ≥110 mmHg) (2). Similarly, according to the 2017 ACC/AHA guideline, participants were reclassified into four categories: Normal (untreated SBP <120 mmHg and DBP <80 mmHg); Elevated (untreated SBP 120–129 mmHg and/or DBP <80 mmHg); Grade 1 hypertension (SBP 130–139 mmHg and/or DBP 80–89 mmHg); Grade 2 hypertension (SBP ≥140 mmHg and/or DBP ≥90 mmHg) (1).

### Mortality

Data from the CHNS cohort study were used to estimate the mortality risk from different BP categories defined by ACC/AHA and Chinese guidelines. In the present study, premature death is defined as mortality before age 73.64 years in men and 79.43 years in women, which was the average life expectancies in China in 2015 (21). To overcome the issues related to the arbitrary selection of age threshold for premature mortality, we also adopted the definition from the Global Burden of Disease (GBD) study, where a death that occurred before the potential maximum life expectancy [refer to the standard life expectancy (SLE)] observed at the age of the person who died. Here, the SLE is intended to represent the potential achievable human life spans of an individual at a given age. It is calculated based on the highest national life expectancy projected for the year 2050 (22).

### Assessment of Covariables

Body mass index (BMI) was calculated as weight in kilograms divided by height in meters squared. Some other variables included educational level (illiterate, primary, middle/high school, bachelor, or above), marital status (married, never), residence status (rural, urban), smoking status (non-smoker, current smoker, ex-smoker), drinking status (non-drinker, drinker), self-reported health status in excellent/very good condition (yes, no), and self-reported CVD (yes, no), diabetes (yes, no), cancer (yes, no).

### Statistical Analysis

The percentage and number (95% confidence interval [CI]) of adults with hypertension, recommended antihypertensive treatment and control status of hypertension based on 2017 ACC/AHA guidelines only, 2018 CHL guidelines only, and both or either guideline was estimated using CHARLS sampling weights to extrapolate to Chinese population (≥45 years).

Baseline characteristics of participants from CHNS were compared between hypertension and non-hypertension groups by *t-*tests for continuous variables expressed as mean (standard deviation) or Chi-square tests for categorical variables expressed as *n* (%). Kaplan–Meier (KM) curves were employed to estimate the survival probabilities across different hypertension categories and compared by the log-rank test. The incidence rate (per 1,000 person-years) for all-cause mortality and premature death was calculated. The hazard ratios (HRs) and 95% CIs of death among different BP categories were estimated by Cox proportional hazards model. To test the reliability of our analysis, we fitted three models, with the first model adjusting for age and gender (model 1). The second model included the variables model 1 plus educational level, marital status, rural residents, drinking, or smoking status (model 2). The third model included the variables in model 2 plus BMI, history of diabetes mellitus, CVD, or cancer (model 3). *p*-values for trends were calculated using the quartile median values of BP categories. To validate the association between BP with premature mortality, we repeated the main analyses but redefined the premature death by the GBD approach.

All *p*-values are two-sided and statistical analyses were done with Stata 15.0.

## Results

### Percentage and Number of Hypertension, Recommended Initiating Antihypertensive Medication, Above-The-Goal BP Defined by Chinese and ACC/AHA Guidelines

**Figure 1:**
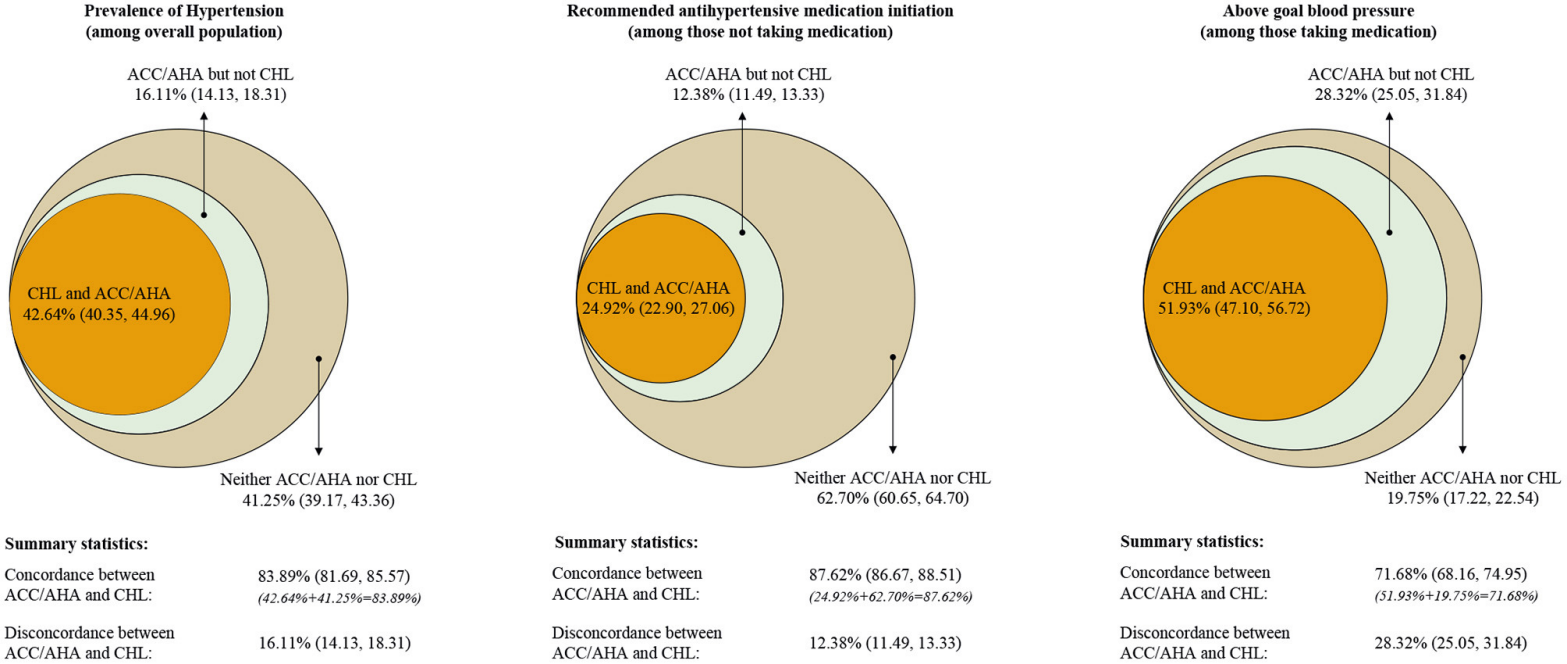
Percentage of hypertension, recommended to initiate antihypertensive medication, above-the goal blood pressure. Data used for estimating was from CHARLS 2011–2012 baseline survey. (Left) Percentage of Chinese adults who have hypertension; (Middle) percentage of Chinese adults who are recommended to initiate antihypertensive medication among those not taking antihypertensive medication; (Right) percentage of Chinese adults with above the goal blood pressure among those taking antihypertensive medication.

**Figure 2:**
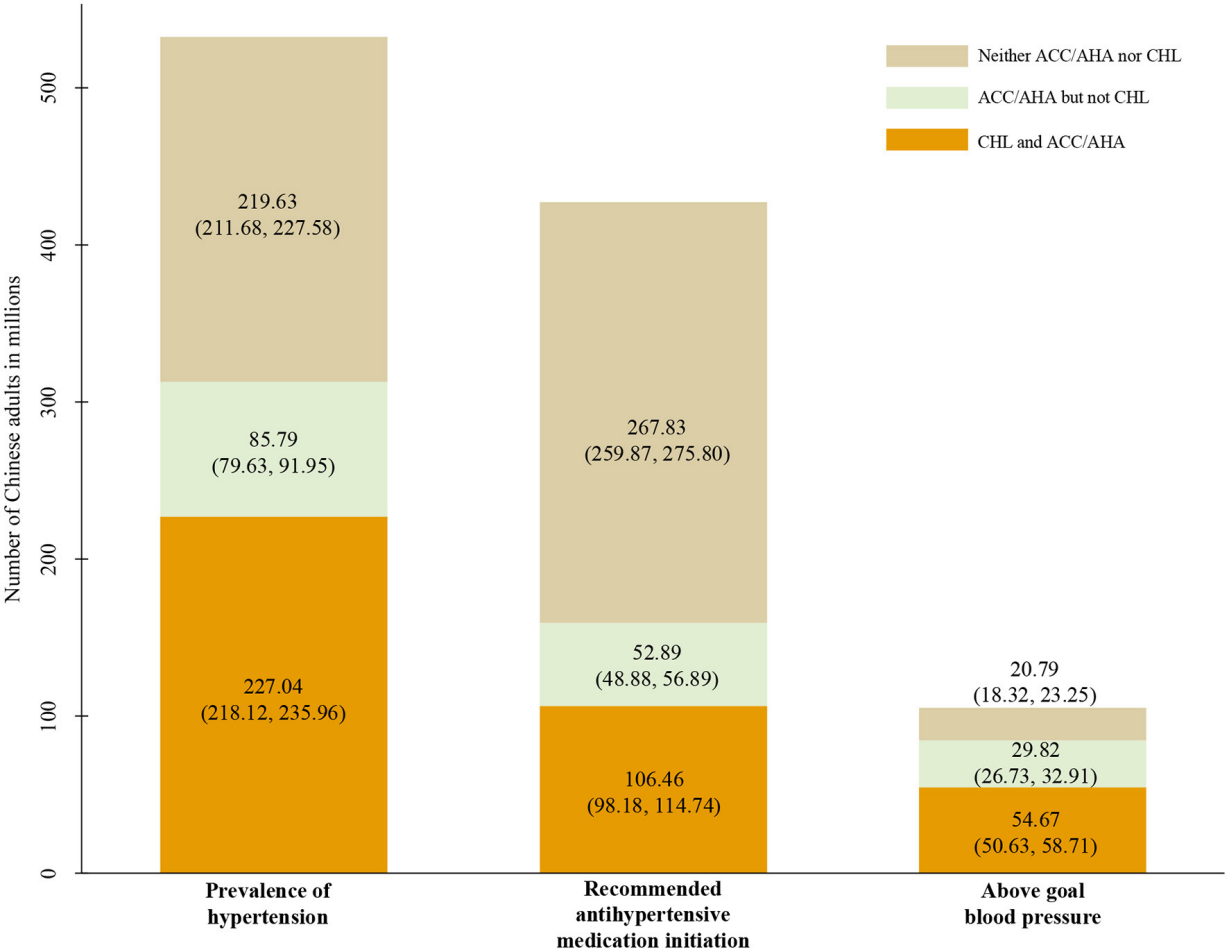
Number of hypertension cases, recommended to initiate antihypertensive medication, above the goal blood pressure. Data used for estimating were collected from CHARLS 2011–2012 baseline survey.

Our current analysis was based on 13,704 subjects after excluding 4,541 subjects who did not participate in the physical examination with missing BP. Overall, we estimated 42.64% (95%CI: 40.35, 44.96; representing 227.04 million) of Chinese adults aged ≥45 years had hypertension according to the CHL, but this would be 16.11% higher (14.13, 18.31; representing 85.79 million) if ACC/AHA guidelines were adopted. Also, 41.25% (39.17, 43.36; representing 219.63 million) did not have hypertension according to either definition. As such, the overall concordance in hypertension status was 83.89% (81.69, 85.57) (Figure 1—Left; Figure 2 and Appendix Table 2 in the Supplementary Material).

Among participants who reported not taking antihypertensive medication (*N* = 11,126), 12.38% (11.49, 13.33; representing 52.89 million) were recommended antihypertensive medication by 2017 ACC/AHA guidelines only. However, 62.70% (60.65, 64.70; representing 267.83 million) were not recommended to initiate antihypertensive medication by either ACC/AHA or CHL guidelines and 24.92% (22.90, 27.06; representing 106.46 million) were recommended to initiate treatment by both guidelines (overall concordance 87.62% [86.67, 88.51]) (Figure 1—Middle; Figure 2 and Appendix Table 2 in the Supplementary Material).

Among those with hypertension taking antihypertensive medication (*N* = 2,578), 28.32% (25.05, 31.84; representing 29.82 million) and 51.93% (47.10, 56.72; representing 54.67 million), 19.75% (17.22, 22.54; representing 20.79 million) was above the ACC/AHA goal only, both and neither guideline, respectively (overall concordance 71.68% [68.16, 74.95]) (Figure 1—Right; Figure 2 and Appendix Table 2 in the Supplementary Material).

### Baseline Information of Participants Included in CHNS Study

A total of 23,063 participants were included in the present analysis. The mean age was 41.08 (range: 18–75) years; 45.24% were male. Over a median of 6.06 years of follow-up, there were 1,673 deaths. Of these deaths, 74.06% (1,239/1,673) were defined as premature. The prevalence of hypertension was 20.22% (4,663/23,063) by CHL definition and 46.85% (10,805/23,063) by ACC/AHA definition. Subjects with hypertension by both definitions tended to be older, received lower education, had a higher proportion of smoking and drinking, higher BMI levels, higher prevalence of CVD, diabetes, and cancer (Appendix Table 3 in the Supplementary Material).

### Associations Between Hypertension Categories and Mortality

**Figure 3:**
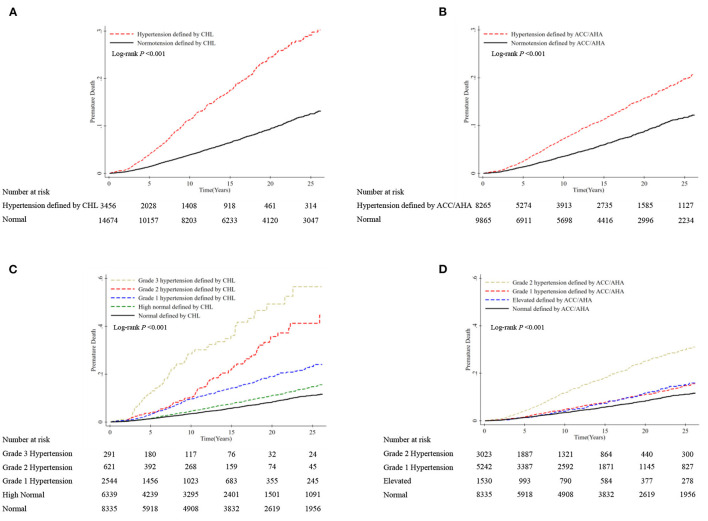
Cumulative incidence of premature mortality according to hypertension categories by CHL and ACC/AHA definition. Premature death is defined as deaths before the age of 73.64 years in men and 79.43 years in women. **(A)** Hypertension was defined as SBP ≥140 mmHg, DBP ≥90 mmHg, or taking antihypertensive medication according to the CHL guidelines; **(B)** hypertension was defined as SBP ≥130 mmHg, DBP ≥80 mmHg, or taking antihypertensive medication according to the 2017 ACC/AHA guidelines; **(C)** participants were categorized as having normal BP (untreated SBP <120 mmHg and DBP <80 mmHg); high normal BP (untreated SBP 120–139 mmHg and/or DBP 80–89 mmHg); Grade 1 hypertension (SBP 140–159 mmHg and/or DBP 90–99 mmHg); Grade 2 hypertension (SBP 160–179 mmHg and/or DBP 100–109 mmHg); and Grade 3 hypertension (SBP ≥180 mmHg and/or DBP ≥110 mmHg) according to the CHL guidelines; **(D)** participants were categorized as having normal BP (untreated SBP <120 mmHg and DBP <80 mmHg); elevated BP (untreated SBP 120–129 mmHg and/or DBP <80 mmHg); Grade 1 hypertension (SBP 130–139 mmHg and/or DBP 80–89 mmHg); and Grade 2 hypertension (SBP ≥140 mmHg and/or DBP ≥90 mmHg) according to the 2017 ACC/AHA guidelines.

Kaplan–Meier curves indicated that the cumulative rate of premature mortality was significantly different between participants with and without hypertension defined by CHL (*P* < 0.001) in Figure 3A. A similar result was found for hypertension defined by ACC/AHA in Figure 3B. However, we could graphically identify that the difference in the cumulative rate between hypertension and non-hypertension groups defined by ACC/AHA was smaller than those defined by CHL. This was supported by the adjusted Cox regression model. We found that hypertension by CHL significantly increased the risk of premature death by 75% (Model 3: 1.75, 1.50–2.04), whereas this risk reduced to 46% (Model 3: 1.46, 1.26–1.69) for hypertension by ACC/AHA (Table 1).

**Table 1:** Hazard ratios of all-cause mortality and premature mortality according to hypertension categories by Chinese Hypertension League (CHL) and European Society of Cardiology (ESC)/European Society of Hypertension (ESH) and American College of Cardiology (ACC)/American Heart Association (AHA) definitions^a,b^.

	***N***	**Death or premature**	**Incidence rate per 1,000**	Model 1*		**Model 2^†^**		**Model 3^‡^**	
			**Person-years**	**HR**	**95%CI**	**HR**	**95%CI**	**HR**	**95%CI**
**ALL-CAUSE MORTALITY**									
**CHL** ^**a**^									
Normal	18,400	1,113	5.75	Reference		Reference		Reference	
Hypertension	4,663	560	16.10	1.35	1.21–1.50	1.49	1.33–1.69	1.65	1.45–1.87
*P* for trend				<0.001		<0.001		<0.001	
**CHL^a^**									
Normal	10,229	574	4.98	Reference		Reference		Reference	
High Normal	8,171	539	6.90	1.03	0.92–1.16	1.05	0.90–1.21	1.12	0.97–1.30
Grade 1 Hypertension	3,453	330	12.91	1.17	1.02–1.35	1.31	1.11–1.54	1.49	1.26–1.77
Grade 2 Hypertension	833	140	22.05	1.61	1.33–1.95	1.78	1.45–2.20	2.07	1.66–2.57
Grade 3 Hypertension	377	90	31.38	2.35	1.87–2.96	2.46	1.92–3.15	2.96	2.30–3.81
*P* for trend				<0.001		<0.001		<0.001	
**ACC/AHA^b^**									
Normal	12,258	716	5.33	Reference		Reference		Reference	
Hypertension	10,805	957	10.18	1.15	1.04–1.27	1.28	1.14–1.44	1.39	1.23–1.57
*P* for trend				<0.001		<0.001		<0.001	
**ACC/AHA^b^**									
Normal	10,229	574	4.98	Reference		Reference		Reference	
Elevated	2,029	142	7.50	1.11	0.92–1.33	1.03	0.82–1.30	1.08	0.86–1.35
Grade 1 Hypertension	6,801	427	6.87	0.99	0.88–1.13	1.06	0.91–1.23	1.14	0.97–1.33
Grade 2 Hypertension	4,004	530	16.68	1.42	1.25–1.60	1.56	1.35–1.80	1.78	1.54–2.07
*P* for trend				<0.001		<0.001		<0.001	
**PREMATURE DEATH^c^**									
**CHL^a^**									
Normal	18,400	865	4.47	Reference		Reference		Reference	
Hypertension	4,663	374	10.75	1.40	1.23–1.59	1.57	1.36–1.82	1.75	1.50–2.04
*P* for trend				<0.001		<0.001		<0.001	
**CHL^a^**									
Normal	10,229	463	4.02	Reference		Reference		Reference	
High Normal	8,171	402	5.15	1.03	0.90–1.18	1.01	0.85–1.21	1.10	0.92–1.32
Grade 1 Hypertension	3,453	218	8.53	1.18	0.99–1.40	1.33	1.09–1.62	1.54	1.25–1.89
Grade 2 Hypertension	833	87	13.70	1.60	1.26–2.04	1.71	1.31–2.24	2.01	1.52–2.66
Grade 3 Hypertension	377	69	24.06	2.90	2.23–3.76	3.06	2.31–4.07	3.76	2.81–5.04
*P* for trend				<0.001		<0.001		<0.001	
**ACC/AHA^b^**									
Normal	12,258	561	4.18	Reference		Reference		Reference	
Hypertension	10,805	678	7.22	1.18	1.05–1.33	1.33	1.15–1.53	1.46	1.26–1.69
*P* for trend				<0.001		<0.001		<0.001	
**ACC/AHA^b^**									
Normal	10,229	463	4.02	Reference		Reference		Reference	
Elevated	2,029	98	5.18	1.03	0.83–1.29	0.90	0.68–1.21	0.96	0.71–1.28
Grade 1 Hypertension	6,801	322	5.18	1.01	0.87–1.17	1.05	0.88–1.26	1.15	0.95–1.38
Grade 2 Hypertension	4,004	356	11.20	1.47	1.27–1.71	1.62	1.36–1.93	1.89	1.58–2.28
*P* for trend				<0.001		<0.001		<0.001	

a *CHL classification of blood pressure: normal BP (untreated SBP <120 mmHg and DBP <80 mmHg); high normal BP (untreated SBP 120–149 mmHg and/or DBP 80–89 mmHg); Grade 1 hypertension (SBP 140–159 mmHg and/or DBP 90–99 mmHg); Grade 2 hypertension (SBP 160–179 mmHg and/or DBP 100–109 mmHg); Grade 3 hypertension (SBP ≥180 mmHg and/or DBP ≥110)*.

b *2017 ACC/AHA classification of blood pressure: Normal BP (untreated SBP <120 mmHg and DBP <80 mmHg); elevated BP (untreated SBP 120–129 mmHg and/or DBP <80 mmHg); Grade 1 hypertension (SBP 130–139 mmHg and/or DBP 80–89 mmHg); Grade 2 hypertension (SBP ≥140 mmHg and/or DBP ≥90 mmHg)*.

c *Premature death is defined as deaths before the age of 73.64 years in men and 79.43 years in women*.

* *Model 1: adjusted for age, gender*.

† *Model 2: adjusted for age, gender, educational level, marital status, whether rural residents, history of drinking, or smoking*.

‡ *Model 3: adjusted for age, gender, educational level, marital status, whether rural residents, history of drinking or smoking, body mass index, previous history of diabetes mellitus, CVD, or cancer*.

While further exploring the BP categories with premature mortality, we found the curves for each BP categorized by CHL diverged over time but not substantial for ACC/AHA, although differences were significant across hypertension categories from Figures 3C,D (both *P* < 0.001). This observation from KM curves was proved by HRs of premature death in the Cox model. Grade 1 hypertension (Model 3: 1.54, 1.25–1.89), Grade 2 hypertension (Model 3: 2.01, 1.52–2.66), and Grade 3 hypertension (Model 3: 3.76, 2.81–5.04) by CHL could significantly increase the risk of premature mortality over normal BP (<120/80 mmHg), although not for high normal (Model 3: 1.10, 0.92–1.32). On the contrary, we only found Grade 2 hypertension by ACC/AHA reached statistical significance (Model 3: 1.89, 1.58–2.28) but not for Grade 1 (Model 3: 1.15, 0.95–1.38) and elevated BP (Model 3: 0.96, 0.71–1.28), compared with normal BP (<120/80 mmHg) (Table 1).

Like the estimates for premature mortality, we found a weak but significant association with all-cause mortality for hypertension by ACC/AHA (Model 3: 1.39, 1.23–1.57), in comparison with the result by CHL (Model 3: 1.65, 1.45–1.87). Also, a significant association was observed for Grade 1 hypertension by CHL (Model 3: 1.49, 1.26–1.77) but not by ACC/AHA (Model 3: 1.14, 0.97–1.33) (Table 1). This could be supported from our further analyses by SBP or DBP levels, where positive association started from SBP ≥140 or DBP ≥90 (Appendix Table 4 in the Supplementary Material).

Sensitivity analyses for the association with premature mortality defined by the GBD study revealed similar findings as those using age threshold from life expectancy for defining premature mortality, although the percentage of premature mortality differs [99.04% (1,657/1,673) by GBD study vs. 74.06% (1,239/1,673) by the threshold of the expected life year] (Appendix Table 5 in the Supplementary Material).

## Discussion

Our study identified that 2017 ACC/AHA guidelines substantially increased the percentage and number of subjects with hypertension, eligibility for antihypertensive medication compared to 2018 CHL guidelines. Furthermore, we found that the degree of concordance was high between CHL and ACC/AHA guidelines with a few small differences. In addition, we found that hypertension, both by CHL or ACC/AHA definition, would significantly increase the risk of premature death or total death among participants aged 18–75 years. However, this excess risk was diminished for hypertension and not observed for the newly defined Grade 1 hypertension by ACC/AHA.

The lower SBP and DBP levels used to define hypertension and the goal of antihypertensive treatment in the 2017 ACC/AHA is the obvious reason for the substantial increase of the percentage of hypertension and not reaching the goal of antihypertensive treatment. A similar increase was also reported in Bangladesh (23) and Korea (24). Our results also emphasized that a high percentage of Chinese adults was provided identical antihypertensive treatment recommendations by ACC/AHA and CHL guidelines. This is in line with the data reported in adults with diabetes in the United States (25). However, the decision to initiate and intensify antihypertensive medication should consider both the benefit and harm as well as the cost of drugs. Therefore, a careful evaluation of the cost-effectiveness of 2017 ACC/AHA guidelines should be performed to provide evidence for hypertension management, especially in China with a large absolute number of hypertensive subjects. Also, we should note that evidence for developing ACC/AHA guidelines as well as ESC/ESH guidelines are mainly from studies in the Caucasian population, which were interpreted carefully while developing CHL guidelines with limited data among Chinese or Asians.

The positive associations have been repeatedly reported between hypertension with cardiovascular incidence and total mortality among young or middle-aged adults (20, 26–30). This is consistent with our study using the traditional BP threshold for hypertension (140/90 mmHg). However, a few studies have explored whether the excess risk persisted if a lower threshold for hypertension by ACC/AHA was adopted. In our analysis, we found a high risk of total mortality with the newly defined hypertension but not stage 1 hypertension. This finding is generally in line with results from a meta-analysis (31) and a recent pooled analysis among the Chinese population (8). It is noted that our analysis further found that the risk of all-cause mortality significantly increased with higher systolic or diastolic BP, in a dose-dependent manner. However, this increased risk only reached statistical significance from SBP ≥140 or DBP ≥90, respectively. This partly supported our finding on the null association for the lower BP cut points for stage 1 hypertension from ACC/AHA with mortality.

Although death is inevitable, evidence has shown that most premature deaths in young or middle-aged adults could be highly preventable (13). A few studies have assessed the association between BP categories and premature mortality. In our study, we found that 74.06% of all deaths were premature, which is higher than studies from He et al. (14) in China (55.3%) and Nalini et al. (16) in Iran (63.6%). Furthermore, consistent with the findings for the all-cause mortality, our study demonstrated that hypertension would significantly increase the risk of early death with an adjusted HR of 1.75. This was supported by our additional analyses using the GBD definition for premature death. Our study was consistent with a previous study in a sample of 169,871 Chinese adults aged 40 years and older (14). Our study further indicated a moderate but significantly increased risk of premature mortality with the lower BP criterion for hypertension by ACC/AHA and a linear trend with the increasing SBP and DBP. Meanwhile, we also found that this positive relationship could not be observed for the newly defined stage 1 hypertension.

The limitations of our study need to be acknowledged. First, our study did not report the association between hypertension with cause-specific mortality, particularly cardiovascular death. This will make the interpretation of hypertension effect on total mortality not intuitive. However, we believed that selection of all-cause mortality as an endpoint could avoid misclassification issues compared with disease-specific ones. Meanwhile, our examination of total or premature mortality could provide substantial information from a public health perspective. Second, BP was measured at a single visit for CHARLS, which is different from the requirement from both guidelines about the diagnosis of hypertension. Third, some covariates, such as cancer treatments, glucose or lipid level, or nutraceuticals, which have been reported to be associated with participants' health outcomes, were not collected in the CHNS study, thus, we could not include them in our analysis. However, we assessed our estimates by adding the variable sequentially and found that they were insensitive to the variables included.

## Conclusion

In summary, our study indicated a high degree of concordance between CHL and ACC/AHA guidelines in terms of hypertension status and antihypertensive treatment recommendations. Moreover, this study provided evidence of attenuated mortality risk from hypertension and non-significant risk from newly defined Grade 1 hypertension by ACC/AHA.

Therefore, we should pay more attention to the impact of the 2017 ACC/AHA guidelines and carefully consider the need to increase the appropriate use of antihypertensive medication to reduce hypertension-related burden in China.

## Data Availability Statement

The raw data supporting the conclusions of this article will be made available upon application. The two public-access study data can be downloaded from http://charls.pku.edu.cn/index/en.html (For CHARLS) and https://www.cpc.unc.edu/projects/china (for CHNS).

## Ethics Statement

The studies involving human participants were reviewed and approved by Xi'an Jiaotong University Health Science Center. The patients/participants provided their written informed consent to participate in the CHARLS and CHNS study.

## Author Contributions

KC was responsible for drafting the article and for the overall content. HS and QW assisted with the drafting and revision of the article. RS, FY, JY, XY, RQ, ZZ, and ZH provided inputs to the study concept and design, critically reviewed the results of analyses, and reviewed and contributed significantly to article revision. CL and ZW performed the statistical analyses. TC and CL provided full access to the study data as well as study oversight and article revision. All authors contributed to the article and approved the submitted version.

## Conflict of Interest

The authors declare that the research was conducted in the absence of any commercial or financial relationships that could be construed as a potential conflict of interest.

## Publisher's Note

All claims expressed in this article are solely those of the authors and do not necessarily represent those of their affiliated organizations, or those of the publisher, the editors and the reviewers. Any product that may be evaluated in this article, or claim that may be made by its manufacturer, is not guaranteed or endorsed by the publisher.
