# Supplementary material for: Similarities in Hypertension Status but Differences in Mortality Risk: A Comparison of 2017 ACC/AHA and 2018 Chinese Hypertension Guidelines
Source: Front Cardiovasc Med. 2022 Feb 21;9:784433. doi: 10.3389/fcvm.2022.784433 (PMC8898956; doi:10.3389/fcvm.2022.784433)
Supplement: Supplementary file 1 [file Data_Sheet_1.docx]

**SUPPLEMENTAL MATERIAL**

| **Appendix Table 1** **Blood pressure threshold used to define hypertension, recommend antihypertensive medication, and treatment goal according to the 2017 ACC/AHA Guideline and the 2018 Chinese Guideline** | | |
| --- | --- | --- |
| **Characteristic** | **2017 ACC/AHA** | **2018 CHL** |
| **BP thresholds for definition of hypertension, mm Hg** | | |
| SBP | ≥130 | ≥140 |
| DBP | ≥80 | ≥90 |
| **BP thresholds for recommended antihypertensive medication, mm Hg** | | |
| SBP |  |  |
| General population | ≥140 | ≥140 |
| Aged ≥65 years | ≥130 | ≥150 |
| High cardiovascular disease risk^1^ or CKD | ≥130 | ≥140 |
| Diabetes | ≥130 | ≥130 |
| DBP |  |  |
| General population | ≥90 | ≥90 |
| Aged ≥65 years | ≥80 | ≥90 |
| High cardiovascular disease risk^1^ or CKD | ≥80 | ≥90 |
| Diabetes | ≥80 | ≥80 |
| **BP goals of among those taking antihypertensive medication, mm Hg** | | |
| SBP |  |  |
| General population | < 130 | < 140 |
| Aged ≥65 years | < 130 | < 150 |
| Diabetes or HF | < 130 | < 130 |
| DBP, mmHg |  |  |
| General population | < 80 | < 90 |
| Diabetes or HF | < 80 | < 80 |

^1^ High cardiovascular risk in 2017 ACC/AHA is defined as a history of cardiovascular disease (coronary heart disease, stroke or heart failure) or 10-year predicted cardiovascular disease risk of at least 10% using the pooled cohort risk equations. CKD: chronic kidney disease.

| **Appendix Table 2: Percentage of Chinese adults who have hypertension, who are recommended antihypertensive medication initiation and with above goal blood pressure** | | | | | |
| --- | --- | --- | --- | --- | --- |
|  |  |  | 2017 ACC/AHA | | Overall |
| Hypertension^1^ | 2018 CHL |  | Yes | No |  |
|  |  | Yes | 42.64(40.35,44.96) | 0 | 42.64(40.35,44.96) |
|  |  | No | 16.11(14.13,18.31) | 41.25(39.17,43.36) | 57.36(55.04,59.65) |
|  |  | Overall | 58.75(56.64,60.83) | 41.25(39.17,43.36) | 100% |
|  |  | Overall concordance: 83.89(81.69,85.57); Overall discordance: 16.11(14.13,18.31) | | | |
| Recommended antihypertensive medication^2^ |  |  | Yes | No |  |
|  |  | Yes | 24.92(22.90,27.06) | 0 | 24.92(22.90,27.06) |
|  | 2018 CHL | No | 12.38(11.49,13.33) | 62.70(60.65,64.70) | 75.08(72.94,77.10) |
|  |  | Overall | 37.30(35.30,39.35) | 62.70(60.65,64.70) | 100% |
|  |  | Overall concordance: 87.62(86.67,88.51); Overall discordance: 12.38(11.49,13.33) | | | |
| Above goal blood pressure^3^ |  |  | Yes | No |  |
|  | 2018 CHL | Yes | 51.93(47.10,56.72) | 0 | 51.93(47.10,56.72) |
|  |  | No | 28.32(25.05,31.84) | 19.75(17.22,22.54) | 48.07(43.28,52.90) |
|  |  | Overall | 71.68(68.16,74.95) | 19.75(17.22,22.54) | 100% |
|  |  | Overall concordance: 71.68(68.16,74.95); Overall discordance: 28.32(25.05,31.84) | | | |

^1^among all subjects

^2^among those not taking antihypertensive medication

^3^among those taking antihypertensive medication

| **Appendix Table 3. Baseline characteristics of the study population on the presence of hypertension, both by CHL and ACC/AHA definition** | | | | | | | | |
| --- | --- | --- | --- | --- | --- | --- | --- | --- |
|  | Total | CHL guideline | |  |  | ACC/AHA guideline | |  |
|  |  | Non-hypertension | Hypertension^*^ | *P* value |  | Non-hypertension | Hypertension^†^ | *P* value |
| Number of participants | 23063 | 18400 | 4663 |  |  | 12258 | 10805 |  |
| Age mean±SD | 41.08±14.87 | 38.01±13.68 | 53.23±13.03 | <0.001 |  | 36.25±13.25 | 46.57±14.69 | <0.001 |
| Age |  |  |  | <0.001 |  |  |  | <0.001 |
| 18-44 | 14014(60.76) | 12855(69.86) | 1159(24.86) |  |  | 9183(74.91) | 4831(44.71) |  |
| 45-64 | 7283(31.58) | 4742(25.77) | 2541(54.49) |  |  | 2632(21.47) | 4651(43.04) |  |
| 65-75 | 1766(7.66) | 803(4.36) | 963(20.65) |  |  | 443(3.61) | 1323(12.24) |  |
| Gender |  |  |  | <0.001 |  |  |  | <0.001 |
| Male | 10434(45.24) | 7934(43.12) | 2500(53.61) |  |  | 4725(38.55) | 5709(52.84) |  |
| Female | 12629(54.76) | 10466(56.88) | 2163(46.39) |  |  | 7533(61.45) | 5096(47.16) |  |
| Educational level |  |  |  | <0.001 |  |  |  | <0.001 |
| Illiterate | 4775(21.29) | 3567(19.85) | 1208(27.08) |  |  | 2443(20.36) | 2332(22.35) |  |
| Primary school | 4143(18.47) | 3293(18.32) | 850(19.05) |  |  | 2155(17.96) | 1988(19.05) |  |
| Middle/High school | 11522(51.36) | 9443(52.54) | 2079(46.60) |  |  | 6318(52.65) | 5204(49.88) |  |
| Bachelor or above | 1993(8.88) | 1669(9.29) | 324(7.26) |  |  | 2083(9.03) | 910(8.72) |  |
| Marital status |  |  |  | <0.001 |  |  |  | <0.001 |
| Never | 2700(11.85) | 2512(13.83) | 188(4.08) |  |  | 1879(15.54) | 821(7.69) |  |
| Married | 20077(88.15) | 15652(86.17) | 4425(95.92) |  |  | 10215(84.46) | 9862(92.31) |  |
| Registered residence |  |  |  | <0.001 |  |  |  | <0.001 |
| Urban | 9824(42.60) | 7545(41.01) | 2279(48.87) |  |  | 4944(40.33) | 4880(45.16) |  |
| Rural | 13239(57.40) | 10855(58.99) | 2384(51.13) |  |  | 7314(59.67) | 5925(54.84) |  |
| Self-reported health |  |  |  | <0.001 |  |  |  | <0.001 |
| (excellent/very good) |  |  |  |  |  |  |  |  |
| Yes | 14883(64.53) | 11992(65.17) | 2891(62.00) |  |  | 8381(68.37) | 6502(60.18) |  |
| No | 8180(35.47) | 6408(34.83) | 1772(38.00) |  |  | 3877(31.63) | 4303(39.82) |  |
| Smoking status |  |  |  | <0.001 |  |  |  | <0.001 |
| Non-smoker | 12459(70.27) | 9702(72.03) | 2757(64.73) |  |  | 6484(75.40) | 5975(65.45) |  |
| Current smoker | 4903(27.66) | 3572(26.52) | 1331(31.25) |  |  | 2022(23.51) | 2881(31.56) |  |
| Ex-smoker | 367(2.07) | 196(1.46) | 171(4.02) |  |  | 94(1.09) | 273(2.99) |  |
| Drinking status |  |  |  | <0.001 |  |  |  | <0.001 |
| Non-drinker | 11695(65.64) | 9071(67.01) | 2624(61.31) |  |  | 6090(70.50) | 5605(61.07) |  |
| Drinker | 6121(34.36) | 4465(32.99) | 1656(38.69) |  |  | 2548(29.50) | 3573(38.93) |  |
| History of diseases |  |  |  |  |  |  |  |  |
| CVD | 180(0.78) | 48(0.26) | 132(2.83) | <0.001 |  | 26(0.21) | 154(1.43) | <0.001 |
| Diabetes | 434(1.88) | 166(0.90) | 268(5.75) | <0.001 |  | 79(0.64) | 355(3.29) | <0.001 |
| Cancer | 101(0.44) | 58(0.32) | 43(0.92) | <0.001 |  | 35(0.29) | 66(0.61) | <0.001 |
| BMI (Kg/m^2^) mean±SD | 22.77±3.67 | 22.27±3.30 | 24.75±4.33 | <0.001 |  | 21.79±2.95 | 23.89±4.07 | <0.001 |
| SBP (mmHg) mean±SD | 118.65±17.88 | 112.60±11.68 | 142.51±18.11 | <0.001 |  | 107.91±10.09 | 130.82±16.96 | <0.001 |
| DBP (mmHg) mean±SD | 76.63±11.06 | 73.13±8.03 | 90.43±10.57 | <0.001 |  | 69.17±6.47 | 85.10±8.87 | <0.001 |
| Data are n (%) or mean (SD).  *Hypertension was defined as SBP ≥140 mm Hg, DBP ≥90 mm Hg, or taking antihypertensive medication according to 2018 CHL  ^†^Hypertension was defined as SBP ≥130 mm Hg, DBP ≥80 mm Hg, or taking antihypertensive medication according to 2017 ACC/AHA  BMI indicates body mass index; DBP, diastolic blood pressure; and SBP, systolic blood pressure. | | | | | | | | |

| **Appendix table 4. Hazard ratios of all-cause mortality and premature mortality according to blood pressure categories** | | | | | | | | | | | |
| --- | --- | --- | --- | --- | --- | --- | --- | --- | --- | --- | --- |
|  | N | Death or Premature | Incidence Rate Per 1000 | Model 1 | |  | Model 2 | |  | Model 3 | |
|  |  |  | Person-years | HR | 95%CI |  | HR | 95%CI |  | HR | 95%CI |
| **All-cause mortality** |  |  |  |  |  |  |  |  |  |  |  |
| SBP Classification |  |  |  |  |  |  |  |  |  |  |  |
| <120 mm Hg | 18352 | 955 | 6.40 | Reference |  |  | Reference |  |  | Reference |  |
| 120-139 mm Hg | 10096 | 715 | 10.57 | 1.01 | 0.91-1.11 |  | 1.01 | 0.90-1.12 |  | 1.04 | 0.91-1.18 |
| 140-159 mm Hg | 6202 | 645 | 22.70 | 1.16 | 1.04-1.29 |  | 1.19 | 1.06-1.33 |  | 1.36 | 1.19-1.56 |
| 160-179 mm Hg | 1961 | 228 | 27.23 | 1.35 | 1.16-1.56 |  | 1.35 | 1.16-1.58 |  | 1.46 | 1.20-1.78 |
| ≥180 mm Hg | 822 | 112 | 35.70 | 1.91 | 1.57-2.33 |  | 1.84 | 1.49-2.26 |  | 2.29 | 1.78-2.94 |
| *P* for trend |  |  |  | <0.001 |  |  | <0.001 |  |  | <0.001 |  |
| DBP Classification |  |  |  |  |  |  |  |  |  |  |  |
| <80 mm Hg | 24216 | 1254 | 7.42 | Reference |  |  | Reference |  |  | Reference |  |
| 80-89 mm Hg | 12624 | 1061 | 12.92 | 1.11 | 1.02-1.20 |  | 1.14 | 1.04-1.25 |  | 1.11 | 0.99-1.24 |
| 90-99 mm Hg | 4996 | 581 | 19.94 | 1.19 | 1.08-1.32 |  | 1.21 | 1.08-1.34 |  | 1.12 | 0.98-1.28 |
| 100-109 mm Hg | 1382 | 182 | 24.44 | 1.38 | 1.18-1.61 |  | 1.42 | 1.21-1.67 |  | 1.38 | 1.13-1.68 |
| ≥110 mm Hg | 618 | 125 | 35.13 | 1.87 | 1.55-2.25 |  | 1.94 | 1.60-2.35 |  | 1.97 | 1.57-2.47 |
| *P* for trend |  |  |  | <0.001 |  |  | <0.001 |  |  | <0.001 |  |
| **Premature death** |  |  |  |  |  |  |  |  |  |  |  |
| SBP Classification |  |  |  |  |  |  |  |  |  |  |  |
| <120 mm Hg | 18352 | 711 | 4.76 | Reference |  |  | Reference |  |  | Reference |  |
| 120-139 mm Hg | 10096 | 474 | 7.01 | 1.05 | 0.93-1.19 |  | 1.05 | 0.92-1.21 |  | 1.11 | 0.94-1.32 |
| 140-159 mm Hg | 6202 | 377 | 13.27 | 1.22 | 1.07-1.40 |  | 1.27 | 1.10-1.47 |  | 1.59 | 1.32-1.91 |
| 160-179 mm Hg | 1961 | 141 | 16.84 | 1.48 | 1.22-1.79 |  | 1.48 | 1.21-1.80 |  | 1.73 | 1.32-2.25 |
| ≥180 mm Hg | 822 | 80 | 25.50 | 2.33 | 1.84-2.96 |  | 2.22 | 1.73-2.85 |  | 3.12 | 2.28-4.27 |
| *P* for trend |  |  |  | <0.001 |  |  | <0.001 |  |  | <0.001 |  |
| DBP Classification |  |  |  |  |  |  |  |  |  |  |  |
| <80 mm Hg | 24216 | 890 | 5.27 | Reference |  |  | Reference |  |  | Reference |  |
| 80-89 mm Hg | 12624 | 679 | 8.27 | 1.14 | 1.03-1.26 |  | 1.18 | 1.06-1.33 |  | 1.15 | 0.99-1.32 |
| 90-99 mm Hg | 4996 | 336 | 11.53 | 1.22 | 1.07-1.39 |  | 1.25 | 1.09-1.44 |  | 1.14 | 0.95-1.37 |
| 100-109 mm Hg | 1382 | 111 | 14.90 | 1.48 | 1.21-1.81 |  | 1.52 | 1.23-1.88 |  | 1.58 | 1.21-2.08 |
| ≥110 mm Hg | 618 | 97 | 27.26 | 2.58 | 2.08-3.20 |  | 2.74 | 2.20-3.42 |  | 3.05 | 2.33-3.99 |
| *P* for trend |  |  |  | <0.001 |  |  | <0.001 |  |  | <0.001 |  |
| DBP indicates diastolic blood pressure; HR, hazard ratio; and SBP, systolic blood pressure.  Premature death is defined as deaths before the age of 73.64 years in men and 79.43 years in women.  *Model 1: adjusted for age and sex.  ^†^Model 2: adjusted for age, sex, educational level, marital status, whether rural residents, history of drinking or smoking.  ^‡^Model 3: adjusted for age, sex, educational level, marital status, whether rural residents, history of drinking or smoking, body mass index, previous history of diabetes mellitus, stroke or cancer. | | | | | | | | | | | |

| **Appendix table 5. Hazard ratios of premature death according to hypertension categories ^1^** | | | | | | | | | | | |
| --- | --- | --- | --- | --- | --- | --- | --- | --- | --- | --- | --- |
|  | N | Premature Death | Incidence Rate Per 1000 | Model 1* | |  | Model 2^†^ | |  | Model 3^‡^ | |
|  |  |  | Person-years | HR | 95%CI |  | HR | 95%CI |  | HR | 95%CI |
| CHL^2^ |  |  |  |  |  |  |  |  |  |  |  |
| Normal | 18400 | 1101 | 5.69 | Reference |  |  | Reference |  |  | Reference |  |
| Hypertension | 4663 | 556 | 15.99 | 1.40 | 1.23-1.59 |  | 1.57 | 1.36-1.82 |  | 1.75 | 1.50-2.04 |
| *P* for trend |  |  |  | <0.001 |  |  | <0.001 |  |  | <0.001 |  |
| CHL^2^ |  |  |  |  |  |  |  |  |  |  |  |
| Normal | 10229 | 564 | 4.89 | Reference |  |  | Reference |  |  | Reference |  |
| High Normal | 8171 | 537 | 6.87 | 1.03 | 0.90-1.18 |  | 1.01 | 0.85-1.21 |  | 1.10 | 0.92-1.32 |
| Grade 1 Hypertension | 3453 | 327 | 12.79 | 1.18 | 0.99-1.40 |  | 1.33 | 1.09-1.62 |  | 1.54 | 1.26-1.89 |
| Grade 2 Hypertension | 833 | 140 | 22.05 | 1.60 | 1.26-2.04 |  | 1.71 | 1.31-2.24 |  | 2.01 | 1.52-2.66 |
| Grade 3 Hypertension | 377 | 89 | 31.04 | 2.90 | 2.23-3.76 |  | 3.06 | 2.31-4.07 |  | 3.76 | 2.81-5.04 |
| *P* for trend |  |  |  | <0.001 |  |  | <0.001 |  |  | <0.001 |  |
| AHA/ACC^3^ |  |  |  |  |  |  |  |  |  |  |  |
| Normal | 12258 | 706 | 5.26 | Reference |  |  | Reference |  |  | Reference |  |
| Hypertension | 10805 | 951 | 10.12 | 1.18 | 1.05-1.33 |  | 1.33 | 1.15-1.53 |  | 1.46 | 1.26-1.69 |
| *P* for trend |  |  |  | <0.001 |  |  | <0.001 |  |  | <0.001 |  |
| AHA/ACC^3^ |  |  |  |  |  |  |  |  |  |  |  |
| Normal | 10229 | 564 | 4.89 | Reference |  |  | Reference |  |  | Reference |  |
| Elevated | 2029 | 142 | 7.50 | 1.03 | 0.83-1.29 |  | 0.90 | 0.68-1.21 |  | 0.96 | 0.71-1.28 |
| Grade 1 Hypertension | 6801 | 425 | 6.83 | 1.01 | 0.87-1.17 |  | 1.05 | 0.88-1.26 |  | 1.15 | 0.95-1.38 |
| Grade 2 Hypertension | 4004 | 526 | 16.55 | 1.47 | 1.27-1.71 |  | 1.62 | 1.36-1.93 |  | 1.89 | 1.58-2.28 |
| *P* for trend |  |  |  | <0.001 |  |  | <0.001 |  |  | <0.001 |  |
| ^1^Premature death is defined according to GBD study, where a death that occurred before the potential maximum life expectancy observed at the age of the person who died. | | | | | | | | | | | |
| ^2^CHL classification of blood pressure: normal BP (untreated SBP <120 mm Hg and DBP <80 mm Hg); high normal BP (untreated SBP 120-149 mm Hg and/or DBP 80-89 mm Hg); Grade 1 hypertension (SBP 140-159 mm Hg and/or DBP 90-99 mm Hg); Grade 2 hypertension (SBP 160-179 mm Hg and/or DBP 100-109 mm Hg); Grade 3 hypertension (SBP ≥180 mm Hg and/or DBP ≥110). | | | | | | | | | | | |
| ^3^2017 ACC/AHA classification of blood pressure: Normal BP (untreated SBP <120 mm Hg and DBP <80 mm Hg); elevated BP (untreated SBP 120-129 mm Hg and/or DBP <80 mm Hg); grade 1 hypertension (SBP 130-139 mm Hg and/or DBP 80-89 mm Hg); grade 2 hypertension (SBP ≥140 mm Hg or DBP ≥90 mm Hg). | | | | | | | | | | | |
| *Model 1: adjusted for age and sex. | | | | | | | | | | | |
| ^†^Model 2: adjusted for age, sex, educational level, marital status, whether rural residents, history of drinking or smoking. | | | | | | | | | | | |
| ^‡^Model 3: adjusted for age, sex, educational level, marital status, whether rural residents, history of drinking or smoking, body mass index, previous history of diabetes mellitus, stroke or cancer. | | | | | | | | | | | |
